# Healthcare Utilization Survey in the Hybrid Model of the Surveillance for Enteric Fever in India (SEFI) Study: Processes, Monitoring, Results, and Challenges

**DOI:** 10.1093/infdis/jiab371

**Published:** 2021-11-23

**Authors:** Reshma Raju, J Kezia Angelin, Arun S Karthikeyan, Dilesh Kumar, Ranjith Kumar R, Nikhil Sahai, Karthikeyan Ramanujam, Manoj Murhekar, A Elangovan, Prasanna Samuel, Jacob John, Gagandeep Kang

**Affiliations:** 1 Department of Paediatrics, Christian Medical College Vellore, Vellore, India; 2 Department of Gastrointestinal Sciences, Christian Medical College Vellore, Vellore, India; 3 National Institute of Epidemiology, Indian Council of Medical Research, Chennai, India; 4 Department of Community Health, Christian Medical College Vellore, Vellore, India

**Keywords:** Healthcare utilization survey, Hybrid surveillance, Enteric fever, community-based surveillance, monitoring

## Abstract

**Background:**

Lack of reliable data in India drove the “Surveillance of Enteric Fever in India” (SEFI) concept. Hybrid surveillance, combining facility-based surveillance for the crude incidence, and a community-based healthcare utilization survey (HCUS) to calculate the factor needed to arrive at the adjusted incidence, was used in 6 sites. The HCUS aimed to determine the percentage of utilization of study facilities by the catchment population for hospitalizations due to febrile illness.

**Methods:**

Population proportional to size sampling and systematic random sampling, in 2 stages, were used to survey 5000 households per site. Healthcare utilization was assessed.

**Results:**

Febrile illness accounted for 20% of admissions among 137 990 individuals from 30 308 households. Only 9.6%–38.3% of those admitted with febrile illness sought care in the study hospitals. The rate of rural utilization of the private sector for hospitalization was 67.6%. The rate of hospitalization for febrile illness, per 1000 population, ranged from 2.6 in Manali to 9.6 in Anantapur; for 25.8% of the deaths associated with febrile illness, no facility was used before death.

**Conclusions:**

One in 5 hospitalizations were associated with fever. Rural utilization of the private sector for hospitalization due to febrile illness was more than that of the public sector. Healthcare utilization patterns for hospital admissions due to febrile illness varied across sites. A meticulously performed HCUS is pivotal for accurate incidence estimation in a hybrid surveillance.

**Clinical Trials Registration:**

ISRCTN72938224.

Effective surveillance systems are a necessity for controlling communicable diseases. The World Health Organization propounds that “effective communicable disease control relies on effective response systems and effective response systems rely on effective disease surveillance” [1–3]. Surveillance systems require data from healthcare facilities and local communities to detect emerging trends of diseases and generate epidemiological data for national regulators. However, because reliable community-based data are scarce in low-middle-income countries, such as India [4, 5], policy makers are forced to rely on information collected by healthcare facilities [6].

Reliance on facility-based surveillance alone would underestimate the disease burden [3, 7] since not everyone who is ill seeks treatment, especially the underprivileged and the vulnerable, owing to numerous sociocultural and health literacy factors [8]. Of those who do seek treatment, a significant proportion may prefer to rely on private practitioners, traditional healers, or local pharmacies [9, 10], which are scarcely represented in India’s disease surveillance platforms and Health Management Information System, which also has numerous data quality issues [11].

Community-based cohort studies may yield more representative burden estimates but have their limitations too. With existing constraints of trained personnel and finances in low-middle-income countries, genuine efforts to monitor and measure health indicators on a large scale at the community level may be further challenged by language, social, and geographic barriers [6, 12, 13]. Even though community-based surveys, when implemented well, provide a high degree of sensitivity, they avowedly lack the specificity of sophisticated laboratory tests to confirm the diagnoses [13].

A hybrid strategy may be used to bridge the gap between the 2 approaches. Such a model would use a sentinel healthcare facility–based surveillance to calculate a crude incidence rate, which would then be adjusted for cases that used other facilities for healthcare, obtained via annual or biannual healthcare utilization surveys (HCUSs) taking place in the catchment populations of the study facilities [14–19].

This strategy was used by tier 2 of the Surveillance for Enteric Fever in India (SEFI) network to generate data on the incidence and burden of severe enteric fever in India, which remains a significant public health concern in the country. Under this framework, 6 geographically distinct sites across India conducted surveillance for the disease between 2018 and 2020. This article describes the processes, monitoring, results, and challenges of the community-based HCUS at each of these sites as part of the SEFI project. The primary aim of HCUS was to estimate the proportion of the catchment populations at each selected site who had a febrile illness and sought inpatient care at hospitals associated with SEFI’s tier 2 over a period of 12 months, which was subsequently factored into the adjusted incidence estimates of enteric fever by the network.

## METHODS

### Study Setting

The study was designed as part of the SEFI network, a more extensive network of surveillance initiated to obtain the burden of enteric fever in India. SEFI was conceptualized as a multitiered surveillance system covering 18 sites, aimed at estimating the burden of enteric fever at different rural, urban, slum, hilly, and tribal settings across India, with varying designs of study at each of the tiers. Christian Medical College Vellore (CMC Vellore), was the central coordinating team for the network. The second tier followed the hybrid surveillance model, a relatively newly developed, cost-effective, and sustainable approach.

This hybrid surveillance was conducted in secondary hospital settings of 5 rural and 1 urban site and their catchment areas across India, namely, the Rural Development Trust Hospital, Bathalapalli (Anantapur district of Andhra Pradesh); Makunda Christian Leprosy and General Hospital, Bazaricherra (Karimganj district of Assam); Duncan Hospital, Raxaul (East Champaran district of Bihar); Lady Willingdon Hospital, Manali (Kullu district of Himachal Pradesh); Chinchpada Christian Hospital (Nandurbar district of Maharashtra) and Civil Hospital, Sector 45, Chandigarh. All study hospitals were charitable hospitals except the one in Chandigarh, which is was a government facility. The catchment population for these hospitals ranged from 100 000 to 700 000. Site descriptions are summarized in Table 1.

**Table 1.** Summary Statistics of the Healthcare Utilization Survey

Characteristic	Anantapur	Chandigarh	Chinchpada	Makunda	Manali	Raxaul	Overall
Setting	Rural	Urban	Rural tribal	Rural hilly	Rural hilly	Rural	…
Administrative units covered, no. of blocks or sectors	8	1	1	2	1	3	16
Approximate study area covered, km^2^	2478	5	1030	540	102	450	4605
Households, no.							
Total approached	5066	5302	5095	5052	5099	5168	30 782
Participating	5048	5084	5034	5042	5011	5089	30 308
With locked doors	8	122	28	0	32	16	206
Refusing to participate	10	96	33	10	56	63	268
People surveyed, no.	19 162	21 725	23 234	24 408	21 697	27 764	137 990
Household characteristics, no. (%)^a^							
Type of family							
Nuclear	4210 (83.4)	4263 (83.9)	2988 (59.4)	3974 (78.8)	3387 (67.6)	3661 (71.9)	22483 (74.2)
Joint/extended	838 (16.6)	819 (16.1)	2046 (40.6)	1068 (21.2)	1624 (32.4)	1428 (28.1)	7819 (25.8)
SES							
Low	3655 (72.4)	3327 (65.4)	2953 (58.7)	3855 (76.5)	1188 (23.7)	3203 (62.9)	18181 (60)
Middle	1384 (27.4)	1691 (33.3)	2046 (40.6)	1160 (23)	3025 (60.4)	1724 (33.9)	11030 (36.4)
High	9 (0.2)	66 (1.3)	35 (0.7)	27 (0.5)	798 (15.9)	162 (3.2)	1097 (3.6)
Water source							
R/O or purified commercial	3621 (71.7)	1066 (21)	124 (2.5)	5 (0.1)	93 (1.9)	18 (0.4)	4927 (16.2)
Municipal tap (inside dwelling)	199 (3.9)	3560 (70)	251 (5)	175 (3.5)	3059 (61)	95 (1.9)	7339 (24.2)
Municipal tap (common to street)	1180 (23.4)	438 (8.6)	1510 (30)	325 (6.4)	1205 (24)	146 (2.9)	4804 (15.9)
Bore well/tube well	43 (0.9)	12 (0.2)	2957 (58.7)	1358 (26.9)	6 (0.1)	4809 (94.5)	9185 (30.2)
Well (covered or uncovered)	2 (0)	4 (0.1)	143 (2.8)	2141 (42.5)	6 (0.1)	17 (0.3)	2313 (7.6)
Natural source (spring/lake/river)	3 (0.1)	4 (0.1)	49 (1)	1038 (20.6)	642 (12.8)	4 (0.1)	1740 (5.7)
Individual characteristics, no. (%)^b^							
Female sex	9411 (49.1)	10555 (48.6)	11618 (50)	11931 (48.9)	10693 (49.3)	13129 (47.3)	67337 (48.8)
Age ≥15 y	15265 (79.7)	15865 (73)	18324 (78.9)	16219 (66.4)	17079 (78.7)	17377 (62.6)	100129 (72.6)
Marital status							
Married	10715 (55.9)	10775 (49.6)	12786 (55)	10498 (43)	11761 (54.2)	12628 (45.5)	69163 (50.1)
Divorced/separated	63 (0.3)	127 (0.6)	132 (0.6)	166 (0.7)	89 (0.4)	64 (0.2)	641 (0.5)
Never married	7367 (38.4)	10182 (46.9)	9139 (39.3)	12571 (51.5)	8794 (40.5)	14344 (51.7)	62397 (45.2)
Widowed	1017 (5.3)	641 (3)	1177 (5.1)	1173 (4.8)	1053 (4.9)	728 (2.6)	5789 (4.2)
Individuals with health insurance	9129 (47.6)	1149 (5.3)	3609 (15.5)	4936 (20.3)	4187 (19.3)	1981 (7.1)	24991 (18.1)

Abbreviations: RO, reverse osmosis; SES, socioeconomic status.

^a^Denominators for household characteristics are the number of participating households for the site.

^b^Denominators for individual characteristics are the number of individuals surveyed at the site.

### Sampling Process

The tier 2 sites were selected, assuming that they were the dominant providers of inpatient medical care in their catchment areas. The catchment area of each of the study hospitals was defined as the geographically adjoining areas from where the most recent 1000 hospitalizations associated with febrile illness (“febrile hospitalizations”) occurred. Mapping of the catchment required reviewing inpatient hospital records of the previous 24 months in each site. Patient addresses were ordered by distance from the study facility, and the community development blocks that covered >80% of the recent 1000 hospital admissions for febrile illness formed the hospital’s catchment. It was anticipated that about 60% of febrile hospitalizations from the catchment area would occur in the study hospital. The annual incidence of hospitalization for febrile illness in these settings has been previously estimated at 6 per 1000 hospitalizations by the National Sample Survey Office [20].

We calculated that 150 febrile hospitalizations in the catchment population were required to estimate the proportion hospitalized at the study facility with a 10% absolute precision and assuming a design effect of 1.5 for intrafamilial and village level clustering. Thus, we surveyed 25 000 individuals from about 5000 randomly selected households (assuming 5 persons per household) to identify 150 febrile hospitalizations in each site.

The HCUS described here was conducted in the catchment areas between June and October 2019. A 2-stage sampling process was used. In the first stage, a random sample of 100 primary sampling units (wards in urban and villages in rural areas) was selected by probability proportional to size sampling technique at each site. Subsequently, systematic random sampling was used to select households within the clusters. From a random start, 50 households were selected from each of the 100 clusters to obtain 5000 household interviews each from 6 sites.

### Data Collection

After obtaining informed consent, data were collected on socioeconomic and demographic variables, mortality and morbidity profile, and health-seeking behavior based on 2-week and 12-month recall. Data were gathered electronically using Survey Solutions, a survey data capture software developed by the World Bank. The questionnaire was translated into 5 regional languages and then back-translated to check for accuracy. Training modules were developed to train 84 field workers and 12 supervisors (14 field workers and 2 supervisors per site) responsible for data collection. The monitoring and evaluation team were trained separately.

### Monitoring Process

The National Institute of Epidemiology (ICMR-NIE), Chennai, was responsible for the on-site field monitoring and validation of the survey. The intrinsic hierarchical workflow of the software allowed quality checks at different points during data collection: fieldworkers formed the lowest tier (interviewers), and the data they collected were checked by the supervisors (second tier); the headquarters consisted of the ICMR-NIE team who performed a final check on the data collected for any quality issues (Supplementary Figure 1). If any were found at either the supervisor or headquarters level, the forms were rejected until query resolution.

Real-time remote data monitoring was executed for each site by the central team in CMC Vellore based on set performance monitoring metrics (Supplementary Figure 2). The components of the performance monitoring matrix were response rate, time taken for each interview, completeness of data collected, validation by an independent monitor, and comparison with external validator indicators from the National Sample Survey and the 2011 Census of India [20, 21]. The central monitoring team quantitively measured the performance at each site based on performance scores given to the field workers and clusters separately.

Scores for the fieldworkers, which could range from 0 to 1 with 0 the worst performance score and 1 the best, were based on the proportion of completeness of documentation of selected parameters and on the percentage of adequately timed interviews. Similarly, scores for the clusters, which also could range from 0 to 1, were created based on weights given for the documentation of the number of admissions, deaths, births from the 12-month recall period, any reported illness from the 2-week recall period, and their comparison with external national validators, such as the 2011 Census of India and the National Sample Survey 2014 data. Corrective actions were taken if any cluster or fieldworker had a score <0.5 (Supplementary Figure 3).

### Statistical Analysis

Frequencies and percentages were calculated for the sociodemographic variables. Rates of all-cause and febrile illnesses and hospitalizations were calculated per 1000 population. The percentage of the patients admitted for febrile illness who sought care in the study hospitals was calculated for the pediatric age group, for adults, and overall. Socioeconomic status was calculated using a modified form of scale [21]. Logistic regression analysis and χ ^2^ tests were conducted to check for associations. Stata software (version 15) was used for analysis.

## RESULTS

This study surveyed 137 990 individuals from 30 308 households across all 6 sites (Table 1). The demographic characteristic of the surveyed population is described in Table 2. On average, 60% of the families surveyed were of low socioeconomic status. The survey reported a high proportion of households practicing open-air defecation at 3 sites, with 13.9% in Anantapur, 26.8% in Chinchpada, and 41.1% in Raxaul.

**Table 2.** Profile of Illnesses Reported for a 2-Week Recall Period

Characteristics of Reported Illness	Illnesses, No. (%)^a^						
	Anantapur (n = 1744)	Chandigarh (n = 2349)	Chinchpada (n = 1949)	Makunda (n = 4758)	Manali (n = 2435)	Raxaul (n = 6752)	Overall (n = 19 987)
Illnesses per 1000 persons, no.							
Any illness	91	108.1	83.9	194.9	112.2	243.2	144.8
Febrile Illness	31.2	69.9	42.2	89.7	29.1	170.8	77.2
Any-cause illness							
Patient aged ≥15 y	1540 (88.3)	1648 (70.2)	1522 (78.1)	3284 (69.0)	2001 (82.2)	4402 (65.2)	14397 (72)
Female patient	925 (53)	1282 (54.6)	1057 (54.2)	2458 (51.7)	1376 (56.5)	3441 (51)	10 539 (52.7)
Antibiotics used	219 (12.6)	444 (18.9)	205 (10.5)	1325 (27.8)	162 (6.7)	909 (13.5)	3264 (16.3)
Type of illness							
Febrile Illness	597 (34.2)	1518 (64.6)	981 (50.3)	2190 (46.0)	632 (26)	4741 (70.2)	10659 (53.3)
Other medical illness	1091 (62.6)	794 (33.8)	921 (47.3)	2414 (50.7)	1737 (71.3)	1778 (26.3)	8735 (43.7)
Pregnancy related	13 (0.7)	11 (0.5)	11 (0.6)	15 (0.3)	13 (0.5)	25 (0.4)	88 (0.4)
Treatment facility							
Government facility	281 (16.1)	634 (27)	486 (24.9)	597 (12.6)	249 (10.2)	149 (2.2)	2396 (12)
Home remedies/no treatment	24 (1.4)	116 (4.9)	145 (7.4)	245 (5.2)	404 (16.6)	364 (5.4)	1298 (6.5)
Pharmacy	78 (4.5)	209 (8.9)	47 (2.4)	2016 (42.4)	1199 (49.2)	3356 (49.7)	6905 (34.6)
Private facility	941 (54)	915 (39)	1245 (63.9)	1292 (27.2)	400 (16.4)	1806 (26.8)	6599 (33)
Study hospital	103 (5.9)	254 (10.8)	18 (0.9)	239 (5)	86 (3.5)	96 (1.4)	796 (4)
Traditional healers/registered medical practitioners	317 (18.2)	221 (9.4)	8 (0.4)	369 (7.8)	97 (4)	981 (14.5)	1993 (10)
Febrile Illnesses^b^							
* *Patient aged ≥15 y	448 (75)	955 (62.9)	699 (71.3)	1192 (54.4)	414 (65.5)	2669 (56.3)	6377 (59.8)
Female patient	306 (51.3)	789 (52)	523 (53.3)	1144 (52.2)	364 (57.6)	2346 (49.5)	5472 (51.3)
Antibiotics used	131 (21.9)	269 (17.7)	140 (14.3)	1022 (46.7)	87 (13.8)	798 (16.8)	2447 (23)
Treatment facility							
Government facility	110 (18.4)	377 (24.8)	281 (28.6)	309 (14.1)	71 (11.2)	68 (1.4)	1216 (11.4)
Home remedies/no treatment	8 (1.3)	53 (3.5)	50 (5.1)	56 (2.6)	91 (14.4)	151 (3.2)	409 (3.8)
Pharmacy	21 (3.5)	119 (7.8)	9 (0.9)	1108 (50.6)	303 (47.9)	2583 (54.5)	4143 (38.9)
Private facility	306 (51.3)	705 (46.4)	627 (63.9)	556 (25.4)	105 (16.6)	1084 (22.9)	3383 (31.7)
Study hospital	37 (6.2)	131 (8.6)	8 (0.8)	72 (3.3)	30 (4.7)	51 (1.1)	329 (3.1)
Traditional healers/registered medical practitioners	115 (19.3)	133 (8.8)	6 (0.6)	89 (4.1)	32 (5.1)	804 (17)	1179 (11.1)

^a^Data represent no. (%) of illnesses unless otherwise specified.

^b^Denominators in the febrile illness section are the number of febrile illnesses reported for the site for the 2-week recall period.

From a 2-week recall, the rate of any-cause illness ranged from 83.9 to 243.2 per 1000 persons across the sites. The populations in Manali (49.2%), Makunda (42.4%), and Raxaul (49.7%) often relied on pharmacies for the treatment of illnesses that occurred in the 2-week recall period, while private clinics or hospitals were most often used in Anantapur (54%), Chandigarh (39%), and Chinchpada (63.9%). Within the same recall period, the rate of febrile illnesses ranged from 29.1 to 170.8 per 1000 persons and constituted 53.3% of all illnesses reported across the sites. The healthcare-seeking patterns for this group were similar to those in persons who reported any illnesses, with only 11.4% seeking care in the government facilities, 38.9% in pharmacies, 31.7% in private facilities, and 3.1% in the study hospitals (Table 2). Self-reported use of antibiotics in febrile illnesses in the 2-week recall period varied across sites, ranging from 13.8% in Manali to 46.7% in Makunda. However, it must be noted that 42% of the population did not know whether they had taken an antibiotic.

This study captured 4184 hospitalization episodes that occurred within a 12-month recall period across all sites, with 864 (20.6%) being for febrile illness (Table 3). The type of facility used for any hospitalization was classified into government and private facilities, and it was observed that most such events occurred at private hospitals in Anantapur (67.8%) and Raxaul (86.3%). In comparison, government hospitals were used more often in Chandigarh (90%), Chinchpada (53.3%), Makunda (50.3%), and Manali (51.5%). In the rural sites, 61.7% used the private sector for any inpatient care. Among all admissions, only 0.65% used health insurance, although 928 (22.2%) reported having some form of health insurance. The most common reasons listed for choosing a particular healthcare facility were the distance from home (48.6%), low cost of treatment (21.6%), quality of care (14.5%), and short waiting periods before treatment (11.5%).

**Table 3.** Profile of Hospitalizations Captured for a 12-Month Recall Period

Characteristics of Reported Hospitalizations	Hospitalizations, No. (%)						
	Anantapur (n = 904)	Chandigarh (n = 693)	Chinchpada (n = 643)	Makunda (n = 740)	Manali (n = 437)	Raxaul (n = 767)	Overall (n = 4184)
Hospitalizations per 1000 persons, no.							
Any cause	47.2	31.9	27.7	30.3	20.1	27.6	30.3
Febrile Illness	9.6	8.7	8	3.9	2.6	5.5	6.3
Any-cause hospitalizations							
Patient aged ≥15 y	823 (91.0)	632 (91.2)	588 (91.4)	681 (92.0)	415 (95.0)	690 (90.0)	3829 (91.5)
Female patient	506 (56.0)	484 (69.8)	415 (64.5)	554 (74.9)	314 (71.8)	469 (61.2)	2742 (65.5)
Government facility	291 (32.2)	463 (66.8)	343 (53.3)	372 (50.3)	225 (51.5)	105 (13.7)	1799 (43.0)
Private facility	466 (51.6)	69 (10.0)	258 (40.1)	105 (14.2)	112 (25.6)	513 (66.9)	1523 (36.4)
Study hospital	147 (16.3)	161 (23.2)	42 (6.5)	263 (35.5)	100 (22.9)	149 (19.4)	862 (20.6)
Type of illness							
Febrile Illness	184 (20.4)	188 (27.1)	187 (29.1)	96 (13.0)	56 (12.8)	153 (20.0)	864 (20.6)
Other medical condition	355 (39.3)	142 (20.5)	145 (22.6)	133 (18.0)	84 (19.2)	228 (29.7)	1087 (26.0)
Surgical and trauma	178 (19.7)	94 (13.6)	83 (12.9)	101 (13.6)	79 (18.1)	201 (26.2)	736 (17.6)
Childbirth related	187 (20.7)	269 (38.8)	228 (35.5)	410 (55.4)	218 (49.9)	185 (24.1)	1497 (35.8)
Febrile Illness hospitalizations^b^							
Patient aged ≥15 y	131 (71.2)	152 (80.9)	152 (81.3)	74 (77.1)	45 (80.4)	110 (71.9)	664 (76.9)
Female patient	88 (47.8)	86 (45.7)	83 (44.4)	39 (40.6)	26 (46.4)	63 (41.2)	385 (44.6)
Government facility	57 (31.0)	103 (54.8)	88 (47.1)	41 (42.7)	25 (44.6)	8 (5.2)	322 (37.3)
Private facility	95 (51.6)	13 (6.9)	81 (43.3)	19 (19.8)	11 (19.6)	109 (71.2)	328 (38.0)
Study hospital	32 (17.4)	72 (38.3)	18 (9.6)	36 (37.5)	20 (35.7)	36 (23.5)	214 (24.8)
Duration of hospitalization for AFI, median, d	4	4	4	4	4	4	4

^a^Data represent no. (%) of hospitalizations unless otherwise specified.

^b^Denominators for AFI hospitalizations are the number of AFI hospitalizations reported for the site for the 12-month recall period.

Across all 6 sites, 864 febrile hospitalization episodes that occurred within a 12-month recall period were captured. The febrile hospitalization rate per 1000 persons reported from hilly areas such as Manali and Makunda was low, with rates of 2.6 and 3.9 respectively, and in the plains, the rates were 5.5 in Raxaul, 8 in Chinchpada, 8.7 in Chandigarh, and 9.6 in Anantapur. Utilization of study hospitals for these events were 9.6% in Chinchpada, 17.4% in Anantapur, 25.5% in Raxaul, 35.7% in Manali, 37.5% in Makunda, and 38.3% in Chandigarh. At the rural sites, 67.6% of febrile hospitalizations occurred in private facilities.

A total of 699 deaths were recorded in the 12-month recall across all sites. They were most often due to a chronic medical condition (35.5%), and a majority occurred in the ≥60-year age group (57.8%). Healthcare-seeking behavior before death varied across sites, with public facilities preferred in Anantapur (44.8%), Chandigarh (83.1%), and Chinchpada (37.3%); no treatment sought or treated at home in Makunda (49%) and Manali (44%); and Raxaul’s population most often choosing to receive treatment at a private facility (47.6%), before death due to any cause. Among the deaths recorded, 13.9% occurred with an associated febrile condition; of persons dying with a febrile illness, 25.8% died without seeking treatment in any facility (Table 4).

**Table 4.** Profile of Deaths in Households, Captured for a 12-Month Recall Period

Characteristics of Reported Deaths	Deaths, No. (%)						
	Anantapur (n = 87)	Chandigarh (n = 77)	Chinchpada (n = 75)	Makunda (n = 157)	Manali (n = 116)	Raxaul (n = 187)	Overall (n = 699)
Age group, y							
0–14	1 (1.1)	6 (7.8)	1 (1.3)	29 (18.4)	2 (1.7)	29 (15.5)	68 (9.7)
15–59	30 (34.5)	28 (36.4)	30 (40.0)	46 (29.3)	47 (40.5)	46 (24.6)	227 (32.5)
≥60	56 (64.4)	43 (55.8)	44 (58.7)	82 (52.2)	67 (57.8)	112 (59.9)	404 (57.8)
Female sex	35 (40.2)	34 (44.2)	26 (34.7)	64 (40.8)	42 (36.2)	80 (42.8)	281 (40.2)
Type of illness preceding death							
Febrile illness	11 (12.6)	12 (15.6)	10 (13.3)	23 (14.6)	8 (6.9)	33 (17.6)	97 (13.9)
Surgical and trauma	4 (4.6)	8 (10.4)	2 (2.7)	3 (1.9)	14 (12.1)	13 (7.0)	44 (6.3)
Other acute medical condition	26 (29.9)	28 (36.4)	11 (14.7)	46 (29.3)	30 (25.9)	41 (21.9)	182 (26.0)
Natural cause (eg, old age)	21 (24.1)	5 (6.5)	25 (33.3)	16 (10.2)	28 (24.1)	33 (17.6)	128 (18.3)
Other chronic medical condition	25 (28.7)	24 (31.2)	27 (36.0)	69 (44.0)	36 (31.0)	67 (35.8)	248 (35.5)
Facility used preceding death due to any cause							
Government facility	39 (44.8)	58 (75.3)	28 (37.3)	59 (37.6)	42 (36.2)	16 (8.6)	242 (34.6)
No treatment/at home	27 (31)	10 (13)	27 (36)	77 (49)	51 (44)	82 (43.9)	274 (39.2)
Private facility	14 (16.1)	3 (3.9)	15 (20)	3 (1.9)	4 (3.5)	62 (33.2)	101 (14.5)
Study hospital	7 (8.1)	6 (7.8)	5 (6.7)	18 (11.5)	19 (16.4)	27 (14.4)	82 (11.7)
Facility used preceding death due to febrile illness							
Government facility	6 (54.6)	9 (75.0)	5 (50.0)	8 (34.8)	4 (50.0)	3 (9.1)	35 (36.1)
No treatment/at home	1 (9.1)	1 (8.3)	2 (20.0)	10 (43.5)	2 (25.0)	9 (27.3)	25 (25.8)
Private facility	2 (18.2)	2 (16.7)	3 (30.0)	0 (0.0)	0 (0.0)	15 (45.4)	22 (22.7)
Study hospital	2 (18.2)	0 (0.0)	0 (0.0)	5 (21.7)	2 (25.0)	6 (18.2)	15 (15.5)

Risk factors associated with febrile hospitalization were analyzed using multivariate logistic regression (Table 5). Age <15 years, belonging to a household of low socioeconomic status, having a child <5 years of age in the family, and having a family size <4 members were associated with febrile hospitalizations.

**Table 5.** Logistic Regression Analysis of Factors Associated With Admissions for Febrile Conditions Among Catchment Populations, Based on a 12-Month Recall Period

Factor	Admissions for Febrile Condition, No.		OR (95% CI)	*P* Value
	Yes	No		
Univariate analysis				
Age, y				
<15	195	37 666	0.81 (.69–.95)	.01
≥15	635	99 494	Reference	
Sex				
Female	379	66 958	Reference	.07
Male	451	70 202	1.13 (.99–1.30)	
SES				
Low	510	77 762	1.22 (1.06–1.40)	.06
Middle or high	320	59 398	Reference	
Child aged <5 y in family				
Yes	288	542	1.03 (.89–1.19)	.40
No	46 698	90 462	Reference	
Family size				
<4 members	383	52 851	1.37 (1.19–1.57)	<.001
≥4 members	447	84 309	Reference	
Multivariate analysis				
Age, y				
<15	195	37 666	0.80 (.68–.95)	.01
≥15	635	99 494	Reference	
Sex				
Female	379	66 958	Reference	.07
Male	451	70 202	1.13 (.99–1.30)	
SES				
Low	510	77 762	1.20 (1.04–1.38)	.01
Middle or high	320	59 398	Reference	
Child aged <5 y in family				
Yes	288	542	1.17 (1.003–1.36)	.046
No	46 698	90 462	Reference	
Family size				
<4 members	383	52 851	1.36 (1.18–1.57)	<.001
≥4 members	447	84 309	Reference	

Abbreviations: CI, confidence interval; OR, odds ratio; SES, socioeconomic status.

### Monitoring

Table 6 summarizes the cluster and fieldworker scores at each of the sites. A cluster score <0.5 was considered to be poor performing. Of the 600 clusters, 131 clusters had scores indicating poor performance and had to be resurveyed under supervision. Cluster scores improved after the resurvey. Mean cluster scores improved from 0.51 to 0.75 in Anantapur, from 0.64 to 0.80 in Makunda, from 0.73 to 0.80 in Chandigarh, from 0.67 to 0.72 in Chinchpada, from 0.58 to 0.62 in Manali, and from 0.71 to 0.75 in Raxaul, after the resurvey. This improvement in scores is statistically significant in all sites except Manali. The mean score for the field workers ranged from 0.64 in Manali to 0.92 in Chinchpada. The field workers who were not performing well were either retrained or replaced. Supplementary Figure 3 represents a sample scoring sheet depicting the performance of each cluster in a site.

**Table 6.** Summary of Cluster Scores and Field Worker Scores Used for Monitoring

Site	Cluster Scores^a^					Low- Scoring Clusters, No.	Field Worker Scores^a^	
	Before Resurvey		After Resurvey		*P* Value			
	Range	Mean (SD)	Range	Mean (SD)			Range	Mean (SD)
Anantapur	0.21–0.73	0.5 (0.14)	0.41–0.92	0.75 (0.13)	<.001	36	0.64–0.95	0.77 (0.08)
Makunda	0.23–0.98	0.64 (0.19)	0.44–0.98	0.80 (0.11)	<.001	27	0.55–0.95	0.77 (0.10)
Chandigarh	0.33–0.96	0.73 (0.14)	0.62–0.96	0.80 (0.08)	<.001	08	0.64–0.93	0.78 (0.07)
Chinchpada	0.37–0.98	0.67 (0.15)	0.42–0.97	0.72 (0.13)	.01	18	0.84–0.99	0.92 (0.04)
Manali	0.22–0.90	0.58 (0.17)	0.23–0.92	0.62 (0.15)	.08	30	0.48–0.77	0.64 (0.08)
Raxaul	0.31–0.92	0.71 (0.15)	0.41–0.92	0.75 (0.11)	.03	12	0.59–0.93	0.77 (0.10)

Abbreviation: SD, standard deviation.

^a^Scores for the fieldworkers—ranging from 0 to 1, with 0 the worst performance score and 1 the best—were based on the proportion of completeness of documentation of selected parameters and on the percentage of adequately timed interviews. Scores for the clusters, also ranging from 0 to 1, were created based on weights given for the documentation of the number of admissions, deaths, births from the 12-month recall, any reported illness from the 2-week recall period, and their comparison with external national validators, such as the 2011 Census of India and the National Sample Survey 2014 data [20, 21].

## Discussion

The utilization of study facilities for febrile hospitalizations at the 6 sites under the SEFI tier 2 study ranged from 9.6% to 38.3%. The SEFI network used these estimates to calculate the adjusted incidence rate for typhoid and paratyphoid fevers in India. The observed utilization rate is lower than the presumed 60% while selecting the facilities for hybrid surveillance. Low and variable utilization of the study facility underscores the importance of serially assessing community-based healthcare utilization to generate adjusted, more accurate estimates of the occurrence of *Salmonella* infections and other diseases associated with fever [4, 7, 18, 19].

High population density, availability of traditional/different systems of medicine in India, the plurality of private health sector providers, which has an almost equal number of qualified doctors and unqualified practitioners, and lack of facilities in the public health sectors could be some of the reasons for low utilization of any single health facility in a particular site [22]. In areas with multiple preferred healthcare facilities, selecting a single sentinel facility for facility-based surveillance in hybrid models may underestimate the incidence rates. Sentinel facilities should be carefully selected because, as the percentage of sentinel facility utilization by the population increases, the uncertainty around the adjustment factor for incidence estimation in hybrid surveillance decreases.

In hilly and rugged terrains, the population’s healthcare-seeking behavior might be different from that of the population in the plains owing to poor accessibility. The febrile hospitalization rates reported from hilly areas such as Manali and Makunda were 2.6 and 3.9 per 1000 persons, respectively, and in the plains, these rates were 5.5 in Raxaul, 8 in Chinchpada, 8.7 in Chandigarh, and 9.6 in Anantapur. In the hybrid model in the Surveillance for Enteric Fever in Asia Project study conducted in Bangladesh, Pakistan, and Nepal, the percentages of all individuals who were reported to have been hospitalized for fever within the past 12 months were 1.3% in Bangladesh, 0.6% in Nepal, and 0.4% in Pakistan [12], comparable to findings in the current study.

Further analysis of healthcare utilization patterns showed that illnesses captured using a 2-week recall period were primarily treated using pharmacies or private sector clinics across all sites. Self-reported antibiotic use in the 2-week recall varied across sites. The proportion of the population who were not aware of whether they consumed an antibiotic during the 2-week recall was alarmingly high. Frequent use of pharmacies as the first point of healthcare in rural areas and unawareness of the medicines consumed is particularly concerning owing to inappropriate antibiotic dispensation by pharmacists, which might worsen the already existing antimicrobial resistance in the community [24, 25]. While tighter regulations might help, they should be accompanied by improved awareness among the pharmacists regarding antibiotic misuse and mass awareness campaigns within communities regarding the risks of unwarranted antibiotic consumption.

In the rural sites, 61.73% of all admissions occurred in a private facility, rising to 67.60% in those with a febrile illness. Overreliance on the private sector for treatment was observed for the illnesses recorded in the 2-week recall period also. This will result in significant out-of-pocket expenditures, ultimately resulting in catastrophic health expenditures and impoverishment of households, continuing the vicious cycle of poverty and ill health, especially in the vulnerable groups [26]. Studies have shown that out-of-pocket expenditures are 4 times higher in the private sector than in public facilities for an episode of hospitalization [27]. Inadequate health insurance coverage (22.18%) and even lower insurance usage (0.65%) during a hospitalization episode further exacerbate this problem. A study done among the urban poor in Delhi reports that only 9.7% were enrolled for the Rashtriya Swasthya Bima Yojana plan [28]. Lack of awareness of health insurance plans that can be availed, poor access, and the limitations of these plans in (eg, in illnesses covered and private hospitals included) may be reasons for underutilization of such plans, as reported in other studies [28].

The study found several risk factors associated with the occurrence of a febrile hospitalization among the catchment populations. Having a child of <5 years of age in the household was one factor, which indicates that this age group may be a driver of infectious diseases in households, as has been previously noted [29–31]. We also found that children <15 years of age had a lower chance of hospitalization due to febrile illness than adults. Other studies have reported delays in treatment seeking among adults, which may be due to inadequate access to healthcare facilities, negligence, and the fear of losing daily wages in rural settings, which leads to more severe disease presentation and hence more hospitalizations [32].

Low socioeconomic status was also associated with febrile hospitalizations, which could be because of financial concerns that delay treatment [33] and the receipt of healthcare from unqualified personnel [34, 35]. Finally, being part of a smaller family (<4 members) increased an individual’s odds (odds ratio, 1.36) of experiencing a febrile hospitalization; this finding , however, was not consistent with those from other studies [36]. Another study reports that crowding was associated with a 60% reduction in the incidence of asthma but had a 2.5-fold increase in the incidence of lower respiratory tract infections [37]. The Surveillance for Enteric Fever in Asia Project reports age, household wealth, and disease severity as important determinants of healthcare seeking for acute febrile illness in Bangladesh, Pakistan, and Nepal [12].

Among the deaths associated with fever, 25.8% occurred at the deceased individual’s home or without treatment. When deaths due to any cause were considered, this percentage rose to 39.2%. Such cases are often unrecorded in the country’s registers, leading to a poor understanding of mortality rates and causes of death in India. Thus, verbal autopsy-based studies, such as the Million Deaths Study [38], remain necessary and should receive continued support in the future.

The current study faced several challenges, such as inclement weather, rugged terrain, language and dialect variations, and differences in sociocultural norms. We also had difficulty finding adequately trained field research assistants who could converse in the local languages in 6 diverse study locations. The survey also had to be halted in Manali for multiple weeks owing to flooding in the area. An observed limitation was recall bias, that is, fewer admission episodes reported beyond 6 months of recall. The investigators also found it extremely challenging to obtain accurate self-reported answers for questions related to the diagnoses, severity of illness, and antibiotic use because of lack of awareness and low literacy rates among respondents from the rural communities of India. Some participants found it difficult to understand what an “antibiotic” was and it was hence difficult to elicit the correct response even after multiple attempts. Lack of knowledge regarding diseases and treatment among the participants make such health surveys difficult in rural India, unlike in developed countries. It was also challenging for the central team to monitor the survey simultaneously in 6 remote locations. This challenge was overcome partly by facilitating on-site monitoring visits by ICMR-NIE, Chennai, and systematic remote monitoring of the performance at each site by CMC Vellore through scoring systems developed for the current study.

In conclusion, the study estimates that 1 in every 5 hospitalizations is for febrile illness. Private facilities are relied on more in rural sites for treatment. The HCUS, in the hybrid surveillance of the tier 2 SEFI study, provided the needed factor to calculate the adjusted incidence of enteric fever in India. HCUSs are an essential component of any hybrid surveillance system to provide accurate estimates of the incidence of illnesses. Rigorously designed HCUSs can help generate reliable epidemiological data for various other febrile illnesses and help track health improvements across a wide variety of settings.

## Supplementary Data

Supplementary materials are available at *The Journal of Infectious Diseases* online. Consisting of data provided by the authors to benefit the reader, the posted materials are not copyedited and are the sole responsibility of the authors, so questions or comments should be addressed to the corresponding author.

jiab371_suppl_Supplementary_Figure_1

jiab371_suppl_Supplementary_Figure_2

jiab371_suppl_Supplementary_Figure_3

jiab371_suppl_Supplementary_Text_HCUS_Protocol

## Notes

***Acknowledgments***. We express our gratitude to the people who participated in the survey, the field workers and supervisors, the research staff at the National Institute of Epidemiology, Chennai, who were involved in the monitoring process, the field staff in the study hospitals of the hybrid surveillance sites, and Swathi Krishna, MD of The Wellcome Trust Research Laboratory, Christian Medical College Vellore, for their support during data collection.

***Author contributions.*** P. S., J. J., and G. K. conceived and designed the study; R. R., A. S. K., K. R., and P. S., and developed the study protocol and analysis plan; R.R. coordinated the project; R. R., M. M., and A. E. coordinated data collection at the sites; R. R., J. K. A., D. K., R. K. R., and N. S. analyzed the data; R. R., N. S., and J. J. drafted the manuscript. All authors have reviewed the manuscript and approved it, had complete access to data, and guaranteed the manuscript.

***Disclaimer.*** The funder had no role in the design analysis, interpretation of the findings, or the plan to publish.

***Financial support.*** This study was part of the National Surveillance System for Enteric Fever in India, supported by the Bill & Melinda Gates Foundation (grant INV-009497-OPP1159351).

***Supplement sponsorship.*** This supplement is sponsored by the Christian Medical College Vellore Association.

***Potential conflicts of interest.*** All authors: No reported conflicts. All authors have submitted the ICMJE Form for Disclosure of Potential Conflicts of Interest. Conflicts that the editors consider relevant to the content of the manuscript have been disclosed.

Presented in part: Asian Conference on Diarrhoeal Disease and Nutrition 2020, Bangladesh, January 2020.
