# Supplementary figures and images for: Healthcare Utilization Survey in the Hybrid Model of the Surveillance for Enteric Fever in India (SEFI) Study: Processes, Monitoring, Results, and Challenges
Source: J Infect Dis. 2021 Nov 23;224(Suppl 5):S529–39. doi: 10.1093/infdis/jiab371 (PMC8914874; doi:10.1093/infdis/jiab371)

**Head quarters**

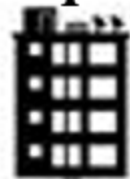

**Supervisors  
and Monitors**

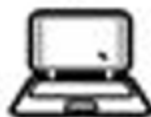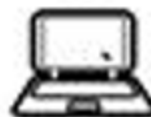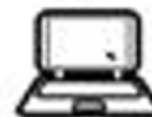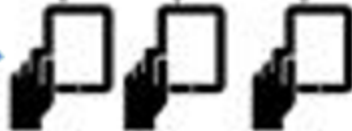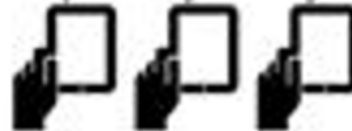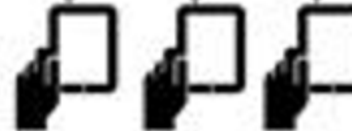

**Interviewers**

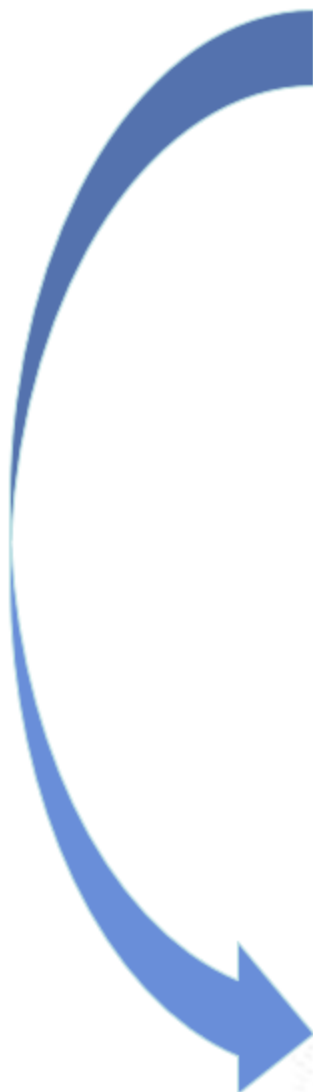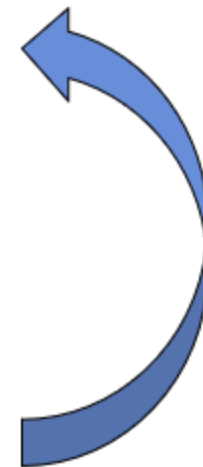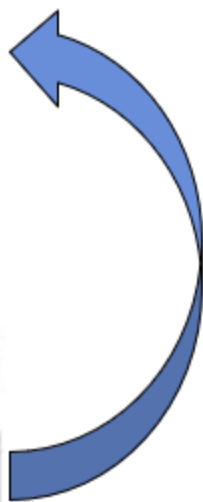

Supplement: jiab371_suppl_Supplementary_Figure_1 [file jiab371_suppl_Supplementary_Figure_1.pdf]

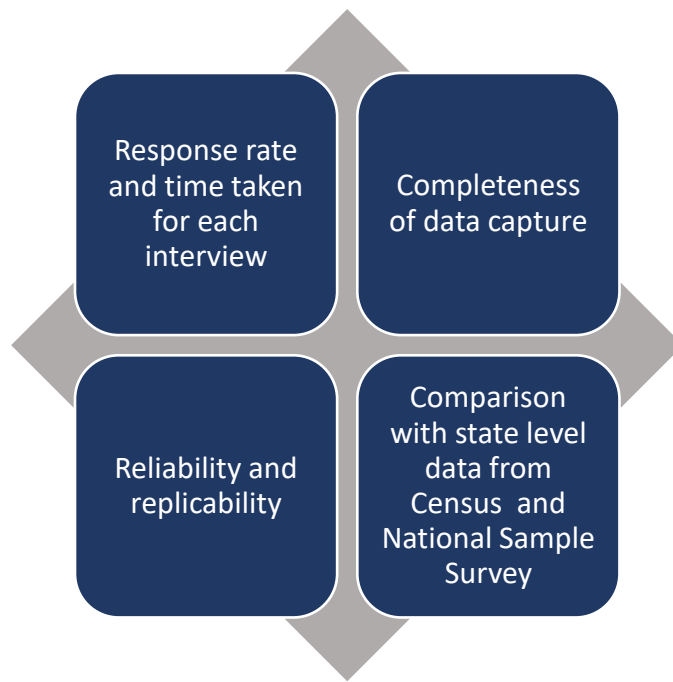

Supplement: jiab371_suppl_Supplementary_Figure_2 [file jiab371_suppl_Supplementary_Figure_2.pdf]
