# Supplementary material for: Healthcare Utilization Survey in the Hybrid Model of the Surveillance for Enteric Fever in India (SEFI) Study: Processes, Monitoring, Results, and Challenges
Source: J Infect Dis. 2021 Nov 23;224(Suppl 5):S529–39. doi: 10.1093/infdis/jiab371 (PMC8914874; doi:10.1093/infdis/jiab371)
Supplement: jiab371_suppl_Supplementary_Figure_3 [file jiab371_suppl_Supplementary_Figure_3.pdf]

1	Cluster_	Int_id	FW SCC	New_Bi	New_d	New_a	New_ill	NEW SC
2	BR086	Bri06	0.845	0.818	1.000	1.000	1.000	0.928
3	BR012	Bri12	0.860	0.633	1.000	1.000	1.000	0.908
4	BR028	Bri11	0.711	0.966	1.000	1.000	1.000	0.908
11	BR048	Bri16	0.593	1.000	1.000	1.000	1.000	0.877
15	BR073	Bri09	0.928	0.910	1.000	0.676	1.000	0.868
21	BR077	Bri13	0.787	0.672	0.728	1.000	1.000	0.855
22	BR010	Bri10	0.871	0.821	1.000	0.712	1.000	0.850
23	BR096	Bri16	0.593	0.787	1.000	1.000	1.000	0.849
24	BR065	Bri01	0.784	0.583	1.000	0.884	1.000	0.844
31	BR017	Bri16	0.593	0.571	1.000	1.000	1.000	0.820
32	BR035	Bri14	0.885	0.731	1.000	0.634	1.000	0.819
36	BR080	Bri16	0.593	0.532	1.000	1.000	1.000	0.815
37	BR055	Bri07	0.757	0.153	1.000	1.000	1.000	0.813
38	BR064	Bri16	0.593	0.511	1.000	1.000	1.000	0.812
39	BR051	Bri03	0.814	0.744	0.538	0.860	1.000	0.806
45	BR032	Bri16	0.593	0.578	1.000	0.877	1.000	0.784
46	BR039	Bri07	0.757	0.168	1.000	0.873	1.000	0.777
47	BR023	Bri04	0.936	0.675	1.000	0.468	1.000	0.777
48	BR074	Bri10	0.871	0.628	1.000	0.545	1.000	0.774
49	BR078	Bri14	0.885	0.513	0.000	1.000	1.000	0.767
56	BR088	Bri08	0.699	0.958	1.000	0.474	1.000	0.746
57	BR067	Bri03	0.814	0.495	0.000	1.000	1.000	0.743
64	BR058	Bri10	0.871	0.734	1.000	0.254	1.000	0.701
65	BR099	Bri13	0.787	0.300	1.000	0.521	1.000	0.698
66	BR044	Bri12	0.860	0.646	0.700	0.420	1.000	0.696
72	BR031	Bri12	0.860	0.394	0.569	0.455	0.922	0.645
77	Br056	Bri08	0.699	0.418	1.000	0.242	1.000	0.604
82	BR042	Bri10	0.871	0.279	0.000	0.483	1.000	0.576
88	BR015	Bri15	0.614	0.648	0.000	0.337	1.000	0.504
89	BR100	Bri03	0.814	0.300	0.651	0.000	1.000	0.504
90	BR050	Bri02	0.674	0.699	0.505	0.000	1.000	0.495
91	BR082	Bri02	0.674	0.408	0.589	0.000	1.000	0.468
92	BR005	Bri05	0.698	0.253	0.000	0.329	0.855	0.456
93	BR018	Bri02	0.674	0.370	0.000	0.214	1.000	0.449

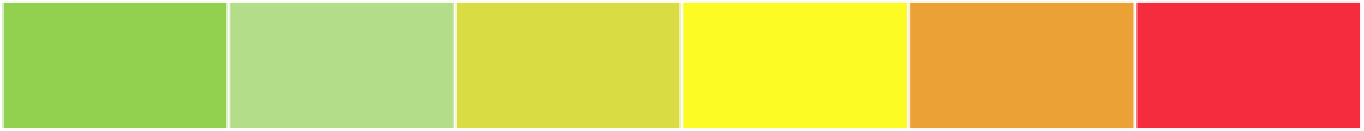

High score Low score
