# Supplementary material for: Healthcare Utilization Survey in the Hybrid Model of the Surveillance for Enteric Fever in India (SEFI) Study: Processes, Monitoring, Results, and Challenges
Source: J Infect Dis. 2021 Nov 23;224(Suppl 5):S529–39. doi: 10.1093/infdis/jiab371 (PMC8914874; doi:10.1093/infdis/jiab371)
Supplement: jiab371_suppl_Supplementary_Text_HCUS_Protocol [file jiab371_suppl_Supplementary_Text_HCUS_Protocol.pdf]

National Surveillance System for Enteric Fever in India

Health utilization survey protocol

Protocol version 2.0

29th May 2019

CHRISTIAN MEDICAL COLLEGE VELLORE

Amendment history

Amendment No.	Version	Date issued	Author of changes	Details of the changes
2	2.0	29/5/2019	Dr Reshma Raju	Defining the catchment area clarified (section 5)
				Definitions included and inclusion criteria added (Section 6)
				Sampling strategy explained in detail (Section 7)
				Corrections made in the data management (Section 10)

Abbreviations

NTAGI	National Technical Advisory Group on Immunization
NSSEFI	National Surveillance System for Enteric Fever in India
NSSO	National Sample Survey Office
PSU	Primary Sampling Unit
SETA	Severe Typhoid Fever Surveillance in Africa
CRF	Case Report Form
CMC	Christian Medical College Vellore
ICH-GCP	International Conference on Harmonization-Good Clinical Practice
ICMJE	International Committee of Medical Journal Editors

1. Background:

Salmonella Typhi is responsible for over 12 million episodes of typhoid fever globally and over 120,000 deaths each year (Crump, Luby and Mintz, 2004; Buckle, Walker and Black, 2012). In South Asia, where access to safe water and sanitation is suboptimal, typhoid is believed to endemic with an estimated average incidence of approximately 4 cases per 1,000 person-years (Buckle, Walker and Black, 2012). Two recently licensed typhoid conjugate vaccines are available for consideration as interventions for the prevention and control of typhoid in India. Although the vaccines are available in the private market and are included in the immunization schedule recommended by the Indian Academy of Pediatrics, the National Technical Advisory Group on Immunization (NTAGI) requires information on the burden of typhoid fever, costs and consequences of disease and the impact of interventions on disease and transmission to make a recommendation on the use of typhoid conjugate vaccines in national immunization programs.

The National Surveillance System for Enteric Fever in India (NSSEFI) was established as a three-tiered surveillance system to address gaps in evidence on the epidemiology of typhoid as identified by India's NTAGI. The tier 1 involves population based active surveillance in four communities across the country. Population based surveillance involves selection of surveillance areas, recruiting all eligible individuals and following them periodically, ideally through home visits to record illness, which is then used to estimate disease incidence. This is an ideal method for calculation of incidence and disease burden estimates (Feikin *et al.*, 2011; Breiman *et al.*, 2012). In addition to being resource intensive and challenging to establish de-novo in settings without an existing health and demographic surveillance system, active surveillance is biased towards the milder illness as treatment is initiated early and appropriately. In order to address the burden of severe disease and to gain insights into disease incidence in rural areas a population-referenced, sentinel hospital surveillance system is being established in tier 2 surveillance deploying methods similar to those described previously (Crump *et al.*, 2003).

The incidence of hospitalised typhoid fever (severe typhoid fever) will be estimated at six locations in smaller towns across rural India in the states of Andhra Pradesh, Maharashtra, Chandigarh, Himachal Pradesh, Bihar and Assam where access to inpatient healthcare is limited to a few service providers. The tier 2 surveillance will primarily target hospitalised typhoid fever and estimate its incidence and not all typhoid fevers presenting to healthcare facilities. The health utilization for outpatient care in these rural population is diverse and only very small proportions seek care for milder illness at the sentinel facilities. This will create significant uncertainties about incidence when the rates are adjusted for those seeking care at alternate facilities. In this approach, the surveillance at the hospital will identify patients with febrile illness requiring hospitals and record their information

in a standardized format. This approach relies on the assumption that individuals with illness of this kind and severity are likely to seek care primarily at the identified facility. While this method is efficient, it is likely to bias the disease burden estimates due to changing health seeking behaviour (Maze *et al.*, 2016).This protocol defines how healthcare utilization patterns in the catchment community of tier 2 sites will be carried out and provides the method for adjustment of incidence estimates to account for those seeking care in hospitalized febrile illness at alternative facilities.

2. Aim:

To estimate the healthcare utilization adjustment factor to be applied for estimating the incidence of hospitalized enteric fever in the hybrid surveillance of the tier 2 of NSSEFI.

3. Objectives:

1. To estimate the proportion of febrile hospitalizations from the catchment that sought inpatient care at the NSSEFI tier 2 sites (based on a 12-month recall).
2. To estimate the proportion of deaths associated with febrile illness (based on a 12-month recall)
3. To identify factors associated with febrile hospitalizations in the catchment population.

4. Methodology:

Two rounds of health utilization surveys will be conducted in the catchment areas of the tier 2 sites. The survey will be conducted twice within a year to account for the variation in healthcare utilization patterns at different times of the year. These tier-2 sites, identified based on their being the dominant inpatient healthcare provider in the area, are

1) Rural Development Trust hospital, Bathalapalli, Andra Pradesh
2) Christian hospital, Chinchipada, Maharashtra
3) Civil hospital sector-45, Chandigarh
4) Lady Willingdon hospital, Manali, Himachal Pradesh
5) The Duncan hospital, Raxaul, Bihar
6) Makunda Christian Leprosy & General hospital, Bazaricherra, Assam.

5. Defining catchment area:

The catchment of each of the above sentinel hospitals will be determined by identifying the geographically contiguous areas from where > 80% of the most recent 960 febrile hospitalizations emerged. The catchment will be demarcated by reviewing inpatient hospital records to identify patients presenting with fever and had a diagnosis compatible with a febrile illness. Assuming 40 febrile hospitalizations each month (based on tier-2 summary data), this would require a review of

about 24 months of inpatient records at most sites. The addresses of these patients will be ordered in terms of distance from the sentinel facility and the area that covers >80% will form the catchment of the hospital. Since at some sites this catchment area may have a very large population, we have decided that the catchment will be truncated at such sites if the total population exceeds 600,000 even if the 80% cut-off is not reached. Similarly, the catchment will be widened, if 80% of cases emerged from a population less than 1,00,000 to ensure that the catchment contains at least a 1,00,000 population. The population of the catchment will be calculated by cumulating the population reported in the most recent census in each village or urban ward that is included in the catchment area and adjusting for annual population growth as estimated from the decennial census. The population of each selected cluster will be verified from the local panchayat or block development offices during the HCUS and the larger estimate will be considered conservatively to be the catchment population.

6. Definitions

- *Definition of household:*

A person or a group of related/unrelated people who live together in the same dwelling unit and share the same kitchen are considered as a household. In some cases, you may find a group of people living together in the same house but each person has separate eating arrangements – in such a case, they should be counted as separate one-person households.

- *Head of the household:*

For the purpose of this survey, head of the household is a person who is recognized as the head by other members in the household. She/he is generally the person who bears the chief responsibility for managing household affairs and takes decisions on behalf of the household. The head of household need not necessarily be the oldest male member or an earning member, but may be a female or a younger member of either sex.

7. Sample size calculation:

Tier 2 sites were selected assuming that they are the dominant providers of inpatient medical care in their catchment. We therefore anticipate that about 60% of febrile hospitalizations from the catchment area will occur in the sentinel hospital. Interviewing 144 febrile hospitalizations will estimate the proportion hospitalized at the study facility with a ±10% precision assuming design effect of 1.5 for intrafamilial and village level clustering. The annual incidence of hospitalization for febrile illness in these settings has been previously estimated at 6/1000 population in national surveys (NSSO Ministry of Statistics and Programme Implementation, India, 2014). Surveying 25,000 individuals from about 5000 randomly selected households (assuming 5 persons per household) will identify 150 febrile hospitalizations from whom health-utilization data can be obtained. Since households are

being selected as a unit of sampling, a natural stratification of similar number of individuals below and above 15 years is likely to be obtained.

8. **Inclusion and exclusion criteria**

Persons included in the survey:

i) All those who normally reside and are present in the household during the entire period of survey – from the first day of survey to the last day of survey (both days inclusive).
ii) All those who were known to be normally residing and had actually stayed in the household during a part of the survey (from the first day of survey to the last day of survey) but were not present at the time of visit of the interviewer.
iii) All those who were known to be normally residing in the household and were not present at the time of visit of the enumerator but are expected to return by the last day of survey.

Persons not included in the survey:

i) Visitors to the household.

9. **Sampling strategy**:

A two-stage cluster sampling strategy will be used for selection of households in the survey. The sampling frame (from 2011 Census) will contain a list of all wards or villages within the catchment area along with approximate number of households in each ward or village. If any village contains less than 100 households, it will be combined with the adjacent village to make one cluster. In the first stage, a random sample of 100 geographical blocks, primary sampling units (PSU) (wards in urban and villages in rural areas) will be selected using probability proportional to size. Since lists of households are unavailable within a cluster, a systematic random sampling will be used to select households within clusters. Selection of the households from each of the cluster will be performed by calculating the total number of households in that cluster and dividing it with the number of households required to be sampled from each cluster to obtain the sampling interval (sampling interval=total no. of households / no. of households required from each cluster). From a random start, 50 households will be selected from each of the 100 clusters to obtain 5000 household interviews at each of the six sites. In each cluster starting from a random point every nth household will be surveyed (based on the sampling interval) till 50 households are surveyed. This will involve two-steps; a method to select the starting point and a method to select successive households from the starting point (sampling interval). If a household is locked or non-responsive, one more visit will be made before replacing it with the nth +1 household. After replacing the non-responsive household, the survey will resume enrolling households selected by the prior schedule. Multifamily tenements should be identified before the start of the interview and sampled individually.

Consent will be obtained from either the head of the house or an adult in the family who will be the key respondent. She/he provide information on all members of the household.

10. Data collection tool:

i) Data collection form required for obtaining a suitable sampling frame: The process starts by collecting few demographic and clinical details such as age, address, date of admission and diagnosis of all fever hospitalizations in the study hospital over the last 24 months. These details along with information such as distance of a particular village and its population which will be obtained from block/panchayat or municipal office, will be used to map the catchment area.

ii) HUS questionnaire: The survey instrument has been adapted from the previously standardized questionnaire used by SETA network. This interviewer administered questionnaire will document survey details (which include village name and cluster number etc.,) household socioeconomic characteristics, household listing of all the residents of the household(which include data on socio-economic, demographic and other variables that affect health utilization as well as typhoid immunization status) , details of any member who was admitted with any illness in the past 2 weeks along with treatment history and cost of illness, a 12-month recall of hospitalization for each member of the household, including diagnosis, place of care and payment options. Additionally, the family will be interviewed about the preferred healthcare facility for inpatient care using a vignette that is applied to two selected household members (youngest child and youngest adult members) in the first round of the survey.

11. Data analysis:

We will estimate the proportion of febrile illness hospitalizations that sought inpatient care at the designated sentinel surveillance hospital, deaths associated with febrile illness and preference for sentinel site as primary healthcare provider with 95% confidence intervals considering the hierarchical nature of data. The proportion of hospitalizations from the catchment population who visit the designated sentinel sites will be used to derive adjustment factors to correct incidence estimates. A probabilistic multiplier model will be used to account for underestimation of hospitalizations in the surveillance sites due to variability in the health utilization patterns. The multiplier is calculated as inverse of the proportion of febrile illness hospitalizations in the surveillance hospitals. To account for uncertainty in multiplication parameter, Monte Carlo approach using a beta distribution will be used to generate 1,000 randomly sampled iterations. Results from this simulation will be used to obtain the median and 95% uncertainty intervals for the number of total cases of febrile hospitalizations in the surveillance hospital. We will also use descriptive statistical methods to describe the socio-demographic characteristics of the survey sample. The proportion of febrile hospitalizations at locations other than surveillance facility will be considered to have a similar

probability of typhoid as those seeking care at the surveillance facility and will be adjusted for in the estimates of incidence. Relationship between socio-demographic baseline factors and utilization of healthcare services at the sentinel surveillance hospital will be assessed using multivariable regression approaches.

12. Data management:

Primary data capture in the first round will be through paper CRFs which will be printed bilingually in English and the appropriate vernacular and entered into an electronic database. The data will be entered onto a local database/server each day after the survey is completed and data quality checks will be performed. Any queries raised along with validation data from supervisors will be reviewed within the next three working days. For the second round, data will be collected by i nterviewers ona tablet, using Survey Solutions ® software from the World Bank and maintained on a secured server instance on AWS. The form filling guidelines will include the questions in regional languages, and these will be standardized with backtranslation.

The dataset will be appropriately backed up and along with completed CRFs, consent forms and logs will be transferred to the central data management team at CMC Vellore within seven working days of completion of the survey at each site. The records will be stored confidentially for a period of 3 years. Deidentified health utilization data will be made accessible to other researchers on request within six months of the primary publications from the NSSEFI.

13. Quality Assurance:

Quality assurance will be integrated into the survey by providing guidelines for data collection, validation and entry. Supervisors will be responsible for carrying out 10% spot checks and back checks of interviews. They will also edit all the CRFs at the field to update errors that have been identified. Research Coordinators will coordinate the data collection at the site, supervise the recruitment of manpower in the state, impart state level training, prepare field movement plan, monitor the quality of entire field work and submit progress report to Project Coordinator. They will also do 5% spot/back checks of the interviews.
Round 2 of the HUS will be monitored by the National Institute of Epidemiology (NIE), Chennai. NIE will be responsible for monitoring the field survey and also for data quality checks.

14. Ethics statement:

The survey will be conducted in compliance with ICH-GCP guidelines. The survey has been approved by the Institutional Review Board at CMC, Vellore [10393 dated 30-11-2016]. All interviews will be conducted after providing eligible participants with a patient information sheet and obtaining a written consent. Any personal identifiers that are collected during the survey for logistics

and quality assurance purposes will not be entered on the database and will be destroyed at the end of the survey cycle.

15. <u>Publications</u>:

Publications from the health utilization surveys will follow the consensus guidelines developed by the investigator group and the ICMJE recommendation for authorship.

References:

Breiman, R. F. *et al.* (2012) 'Population-Based Incidence of Typhoid Fever in an Urban Informal Settlement and a Rural Area in Kenya: Implications for Typhoid Vaccine Use in Africa', *PLOS ONE*, 7(1), p. e29119. doi: 10.1371/journal.pone.0029119.

Buckle, G. C., Walker, C. L. F. and Black, R. E. (2012) 'Typhoid fever and paratyphoid fever: Systematic review to estimate global morbidity and mortality for 2010', *Journal of Global Health*, 2(1). doi: 10.7189/jogh.02.010401.

Crump, J. A. *et al.* (2003) 'Estimating the Incidence of Typhoid Fever and Other Febrile Illnesses in Developing Countries', *Emerging Infectious Diseases*, 9(5), pp. 539–544. doi: 10.3201/eid0905.020428.

Crump, J. A., Luby, S. P. and Mintz, E. D. (2004) 'The global burden of typhoid fever', *Bulletin of the World Health Organization*, 82(5), pp. 346–353.

Feikin, D. R. *et al.* (2011) 'The Burden of Common Infectious Disease Syndromes at the Clinic and Household Level from Population-Based Surveillance in Rural and Urban Kenya', *PLOS ONE*, 6(1), p. e16085. doi: 10.1371/journal.pone.0016085.

Maze, M. J. *et al.* (2016) 'Comparison of the Estimated Incidence of Acute Leptospirosis in the Kilimanjaro Region of Tanzania between 2007–08 and 2012–14', *PLOS Neglected Tropical Diseases*, 10(12), p. e0005165. doi: 10.1371/journal.pntd.0005165.

NSSO Ministry of Statistics and Programme Implementation, India (2014) *Health in India, NSS 71st Round*. Available at: http://mospi.nic.in/sites/default/files/publication_reports/nss_rep574.pdf (Accessed: 30 June 2018).
